# Battery-operated portable PCR system with enhanced stability of Pt RTD

**DOI:** 10.1371/journal.pone.0218571

**Published:** 2019-06-27

**Authors:** Juhun Lim, Sangdo Jeong, Miyoung Kim, Jong-Hyun Lee

**Affiliations:** 1 Department of Biomedical Science and Engineering, Gwangju Institute of Science and Technology (GIST), Gwangju, Republic of Korea; 2 School of Mechanical Engineering, Gwangju Institute of Science and Technology (GIST), Gwangju, Republic of Korea; Texas A&M University College Station, UNITED STATES

## Abstract

This paper reports an outdoor-use polymerase chain reaction (PCR) technology in which stability of resistance temperature detectors (RTDs) is remarkably improved. A thin-film RTD made of non-annealed Pt shows accuracy degradation because the resistance of the RTD tends to decrease during the PCR operation. Thus, the annealing process is applied to the Pt RTD to improve the stability, which is a prerequisite to the accurate measurement of the absolute temperature. Both heaters and the RTD are fabricated on a thin quartz substrate whose melting temperature is high enough for annealing. The performances in the PCR time and power consumption are enhanced by reducing the size of the heater chips with no degradation in the temperature uniformity. A spring-loaded electrode is employed to simplify the procedure of electrical connection to the thermal controller and loading/unloading of the PCR chip. The contact area of the electrical connection is so small that the conductive thermal resistance increases; thereby small heat dissipation can be exploited for low-power operation. The stability of the RTD is experimentally confirmed in terms of resistance variation over repeated PCR operations (four times). The least variation of 0.005%, which corresponds to a negligible temperature variation of 0.038 °C for the PCR, is achieved from the RTD annealed for 5 min at 450 °C. The gel-electrophoresis result indicates that the PCR performance of the proposed system using a film-type PCR chip is comparable to that of a conventional system using a vial tube despite its low power consumption.

## Introduction

Polymerase chain reaction (PCR) is the most widely used method for detecting pathogens such as viruses or bacteria because of its high sensitivity and high selectivity [1–5]. Conventional PCR systems have limitations in that they can only be used in a laboratory environment because of their large size and high power consumption [6, 7]. However, as the need for rapid detection of pathogens in the field is rapidly increasing, studies on portable PCR systems have been actively carried out [8–12]. Most portable PCR devices use a thermocouple as a temperature sensor, which can only measure the relative temperature, not the absolute temperature. This is because the thermocouple measures the temperature difference between the cold junction (reference point) and the hot junction (measuring point) [13–15]. Therefore, there is a disadvantage that the absolute temperature of the cold junction must be measured with temperature sensors such as RTD, thermistor, and integrated circuit transducer to estimate the temperature of the hot junction [16]. As a result, some conventional PCR systems using thermocouples have a limited operable temperature range [17]. In a temperature environment outside the aforementioned range, the measurement error may degrade the PCR performance [16, 18, 19], which frequently occurs in outdoor experiments.

Meanwhile, resistance temperature detectors (RTDs) have a specific resistance value at a certain temperature so that the temperature can be accurately measured with a single calibration. Therefore, an RTD is suitable for outdoor PCRs because it can measure the absolute temperature with no additional compensation. However, thin-film RTDs without annealing show a tendency that the resistance significantly decreases in the repeated thermal cycling [20–21]. This phenomenon is caused by recrystallization of Pt at an elevated temperature, which decreases the thin film resistance [22–24]. As the RTD resistance decreases during the PCR operation, the difference between the measured temperature and the actual temperature becomes large so that recalibrations are essential to perform the PCR properly.

In this study, a Pt RTD sensor was annealed at a temperature much higher than the denaturing temperature to prevent the resistance from additional decreasing during the PCR operation. Two Pt heaters and a Pt RTD were fabricated on a quartz substrate with a small area and thickness to enhance the speed of thermal cycling. The heater was designed to improve the temperature uniformity of the film-type PCR chip using COMSOL simulation. In order to confirm the stability of the annealed Pt RTD experimentally, the RTD resistance was measured after every PCR operation to check whether there was any degradation of the resistance. The amplification performance of the PCR sample (*Escherichia coli* bacteria) was confirmed by gel-electrophoresis and was compared with that of a conventional PCR system using a vial tube.

## Materials and methods

**Fig 1:**
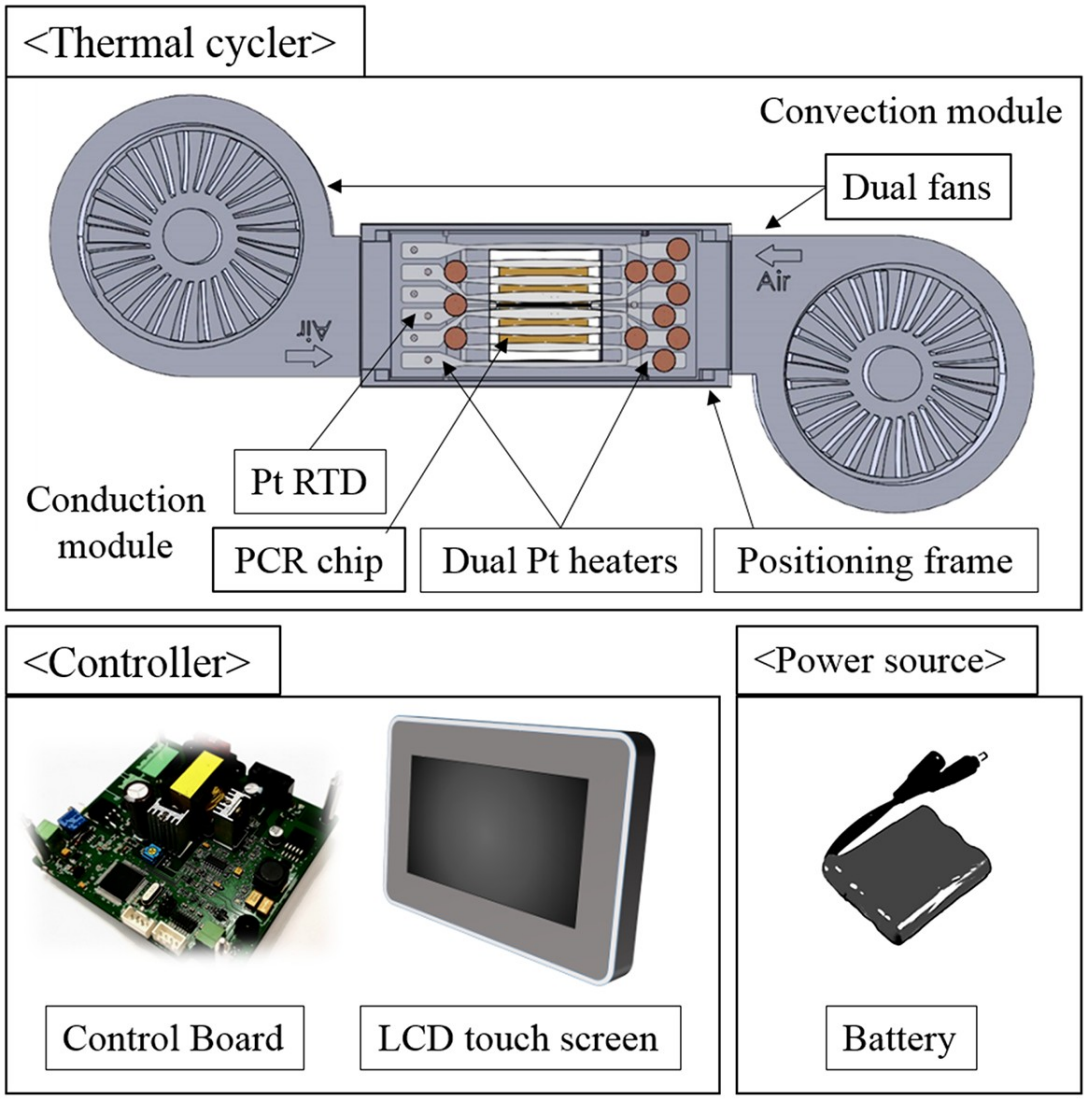
Schematic of the PCR system with a dual heater chip with an RTD, dual fans, a PCR chip, a control board, an LCD touch screen, and a battery.

The entire PCR system consists of a thermal cycler, controller, and power source, as shown in Fig 1. The thermal cycler includes a conduction module (PCR chip, dual Pt heater chip with an RTD sensor, and positioning frame with electrodes) and a convection module (dual cooling fans). The controller consists of a liquid crystal display (LCD) touch screen to set the PCR conditions and display progress and a microcontroller to control thermal cycling by the pulse width modulation (PWM) method. A rechargeable Li-ion battery was used as a power source for portability. In the thermal cycling, Peltier chips, which can perform both heating and cooling, are widely used in the existing PCR system, but they have the disadvantage that the power consumption is as large as 500 W or greater [1]. In this study, we use a Pt thin-film heater and dual cooling fans for low-power operation, which is suitable for portable PCR systems because it consumes only a few watts.

Meanwhile, the use of a film-type PCR chip instead of a PCR vial tube reduced the thermal mass and increased the heat transfer area, thereby improving the temperature rise/fall rate by increasing the heat transfer efficiency [10]. In order to improve the temperature rise/fall rate, the thermal mass of the heater chip was reduced by decreasing the length and thickness of the heater chip substrate (thickness: 0.5 mm, length: 45 mm, and width: 26 mm), as compared to those in the previous work (thickness: 1 mm, length: 76 mm, and width: 26 mm). When reducing the size of the quartz substrate, the heater chip was designed to minimize the thermal mass with the condition that the temperature uniformity is maintained as high as in the previous work. The heat transfer efficiency was further improved by introducing a sandwich structure with a dual heater chip in the upper and lower parts of the PCR chip.

### Heaters and RTD

**Fig 2:**
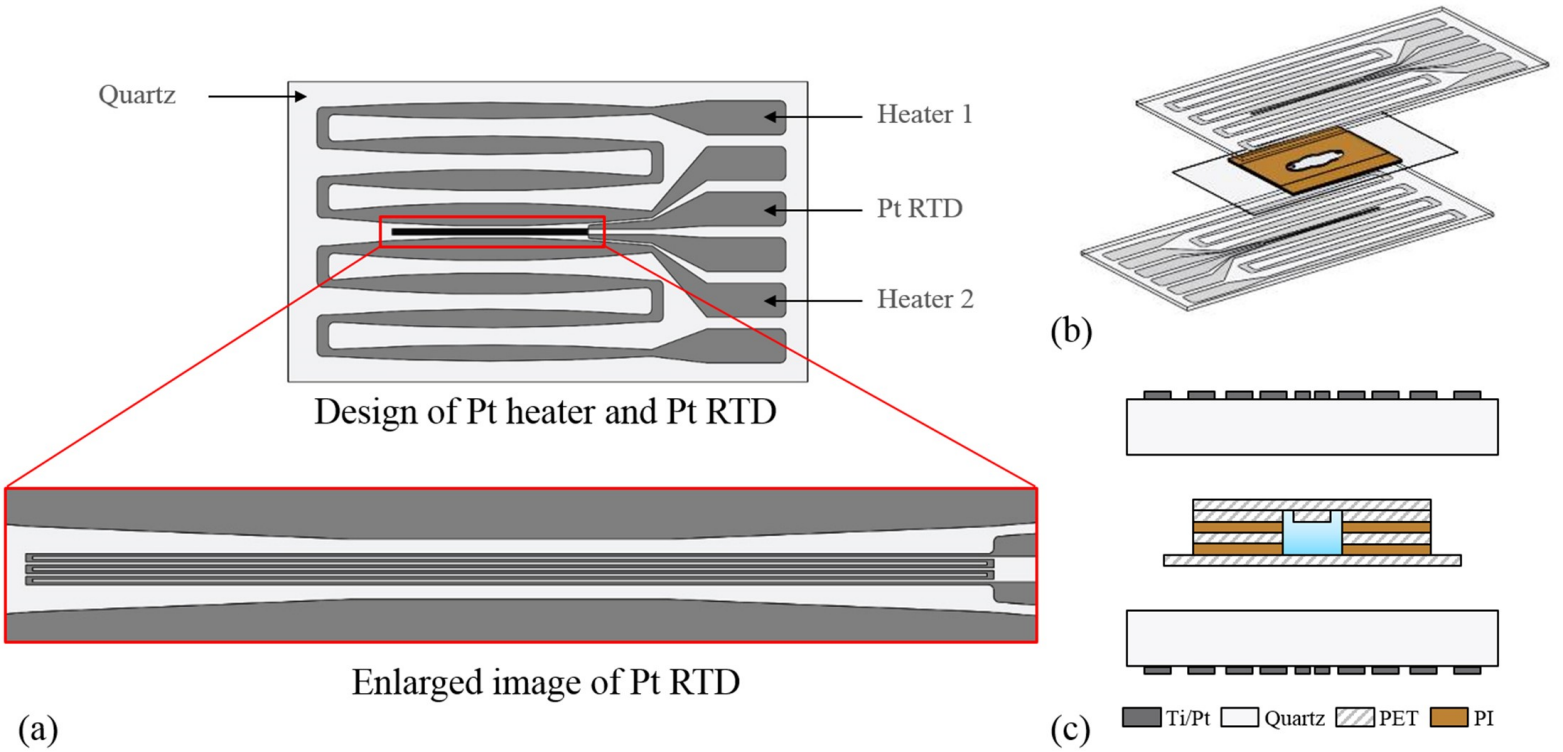
Design of a Pt heater chip with an RTD. Design of Pt heaters and meander pattern RTD (a), sandwiched arrangement between heater chips and a PCR chip (b, c).

When a current is applied to a thin-film Pt heater, a region with a small width (higher resistance) generates more heat than that with a larger width (lower resistance). Thus, the width of the heater pattern should be tapered down from the center to the edge of the heater chip to improve the temperature uniformity throughout the heater chip, as shown in Fig 2(a) [25]. One heater chip contains two electrically isolated heaters to avoid overlapping with the RTD sensor, which allows uniform in-plane temperature distribution. Six straight lines with a width of 50 μm and a length of 16 mm are arranged in a meander pattern to have higher resistance. The length of the RTD is equal to that of the PCR chip, so that the RTD can measure the average temperature of the PCR chip.

Meanwhile, the resistance of the non-annealed Pt thin-film RTD tends to decrease during the PCR operation (58–94 °C), which might lead to degradation in the accuracy of the temperature measurement. Thus, the heater chip with the RTD should be annealed at much higher temperature (for instance, 450–750 °C) than the denaturing temperature (90–100 °C) of the PCR for effective prevention of the RTD from decreasing its resistance. As a substrate of the heater chip, a transparent material such as slide glass is beneficial in that it is possible to employ optical detection for real-time PCR monitoring. However, annealing at such a high temperature may cause deformation of the heater substrate. For example, it is difficult to maintain the stability of slide glass at an annealing temperature of 750 °C because its strain point and softening temperature are 511 °C and 724 °C, respectively [26]. Therefore, quartz, whose strain point and softening temperature are as high as 1120 °C and 1683 °C, respectively [27], was used as the substrate of the heater chip.

Fig 2(b) and 2(c) show a sandwich structure using dual heater chips placed directly on the top and bottom of the PCR chip, which can enhance the temperature uniformity in a vertical direction. The direct contact between the heater chip and the PCR chip will enhance the temperature uniformity in a horizontal direction. This arrangement has the advantages of fast thermal conduction and low convection heat loss, leading to low-power operation.

### PCR chip

**Fig 3:**
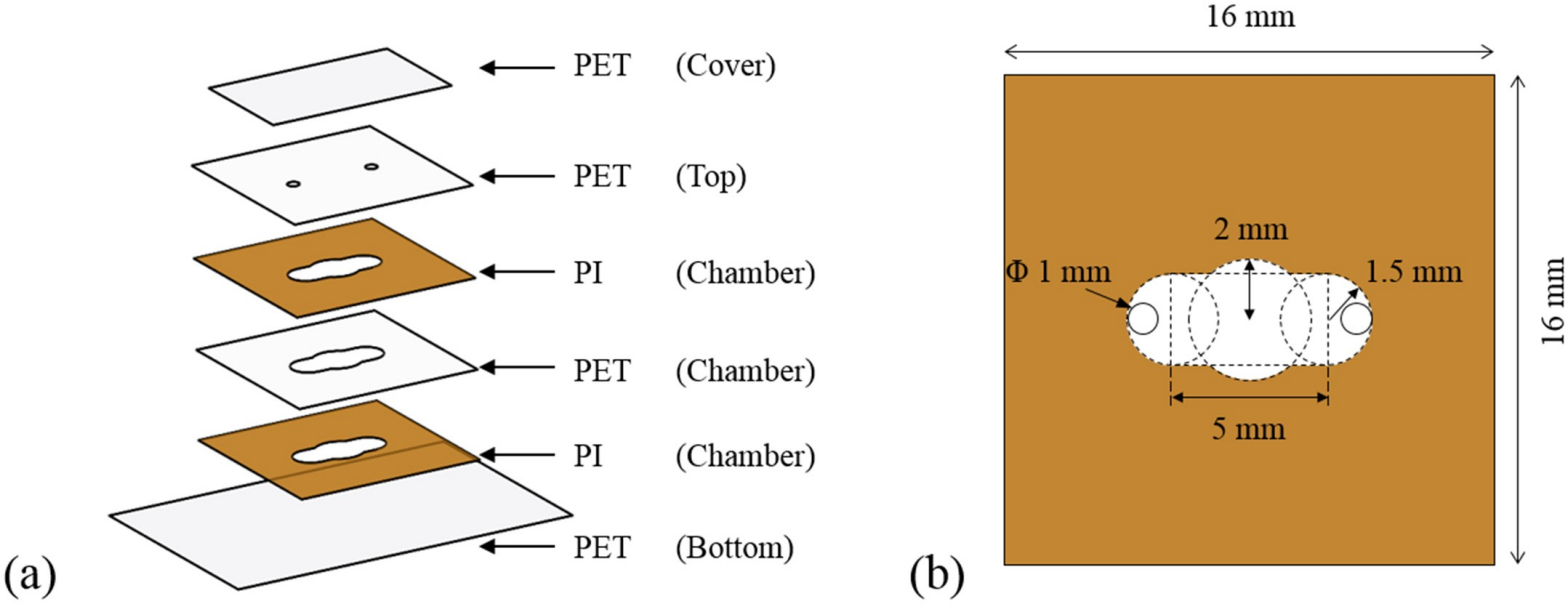
Schematic image of a film-type PCR chip. Exploded layers of the PCR chip (a) and dimensions of the PCR chamber pattern (b).

The disposable PCR chip in this study is made of flexible thin-film polymers, which can enhance the thermal contact and lower the manufacturing cost [28, 29]. Fig 3(a) shows the exploded view of the PCR chip, which consists of bottom, chamber, top, and cover layers. Most layers of the PCR chip were made of transparent PET films to enable the application of fluorescent detection. However, the wall structure of the chamber was made of PI film having a thermal and mechanical stability [30], since PET film is not thermally robust enough to be used as the wall structure of PCR chip. The speed of thermal cycling was further enhanced by reducing the size of the PCR chip, which might decline the temperature uniformity of the PCR chip. The PCR chip was designed to maintain the temperature uniformity on a large area (12.8 mm × 9.9 mm) under ±0.5 °C. Thus, the PCR chip can be used even when the application is extended to multiwells. The detailed dimensions of the PCR chip are described in Table 1. Each film is processed using a cutting plotter (CE6000-40 Plus, Graphtec America, Inc., CA, USA). Fig 3(b) shows the dimension of the chamber, where every corner of the chamber is rounded so that the PCR sample can be filled without air-trap during sample injection. The top layer has an inlet and outlet hole with a diameter of 1 mm.

**Table 1:** Detailed dimensions of the PCR chip with multilayers.

Layers	Material	Dimension [mm^3^]	Structural characteristic	Adhesive layer	Remarks
Cover	PET	10 × 16 × 0.1	rectangular film	Single-sided	sealing cover
Top	PET	16 × 16 × 0.1	rectangular film with two holes (inlet/outlet)	−	−
Chamber	PI	16 × 16 × 0.1	rectangular film with cavity	Double-sided	10*μℓ* chamber volume
	PET	16 × 16 × 0.1	rectangular film with cavity	−	
	PI	16 × 16 × 0.1	rectangular film with cavity	Double-sided	
Bottom	PET	20 × 30 × 0.1	rectangular film	−	−

PI: polyimide, PET: polyethylene terephthalate.

A PCR sample can be injected through the inlet hole using a pipette. The outlet hole is a pathway that allows the initially filled air to escape from the chamber, thereby helping to inject the sample smoothly. Then, the two holes are sealed with an adhesive cover layer (PET film) to prevent the sample from evaporating during the thermal cycling. After completion of the PCR operation, the cover is peeled off to extract the sample with a pipette for gel-electrophoresis.

The use of flat heater chips and thin-film PCR chip can enhance the efficiency of thermal conduction by increasing the surface-to-volume ratio, as compared to vial tubes used in conventional PCR systems. This makes it possible to raise the temperature of the PCR sample quickly even with low-power operation.

### Power source and thermal controller

The PCR system can be operated by a 12 V rechargeable battery (75 mm × 60 mm × 20 mm), which can perform more than 8 times of the PCR operations when it is fully charged. The PCR conditions can be set through the LCD touch screen (Industrial Embedded Computer, 140 mm × 210 mm × 43 mm) equipped with Cortex A8 CPU. The PCR process can be also monitored through the same display. The microcontroller with Atmega128 conducts PWM control for thermal cycling including the measuring temperature from the RTD and operating heaters and fans.

### Electrical contact

An electrical connection is necessary to supply current to the heater and to measure the resistance from the RTD. However, if the electrical connection is made up of soldering, the procedure of replacement becomes complicated because the manual soldering is required every time the heater chip needs to be replaced. In this study, the electrical connection is modified to replace the heater chip easily, allowing the electrode to perform not only as an electrical contact, but also as a physical contact for positioning.

**Fig 4:**
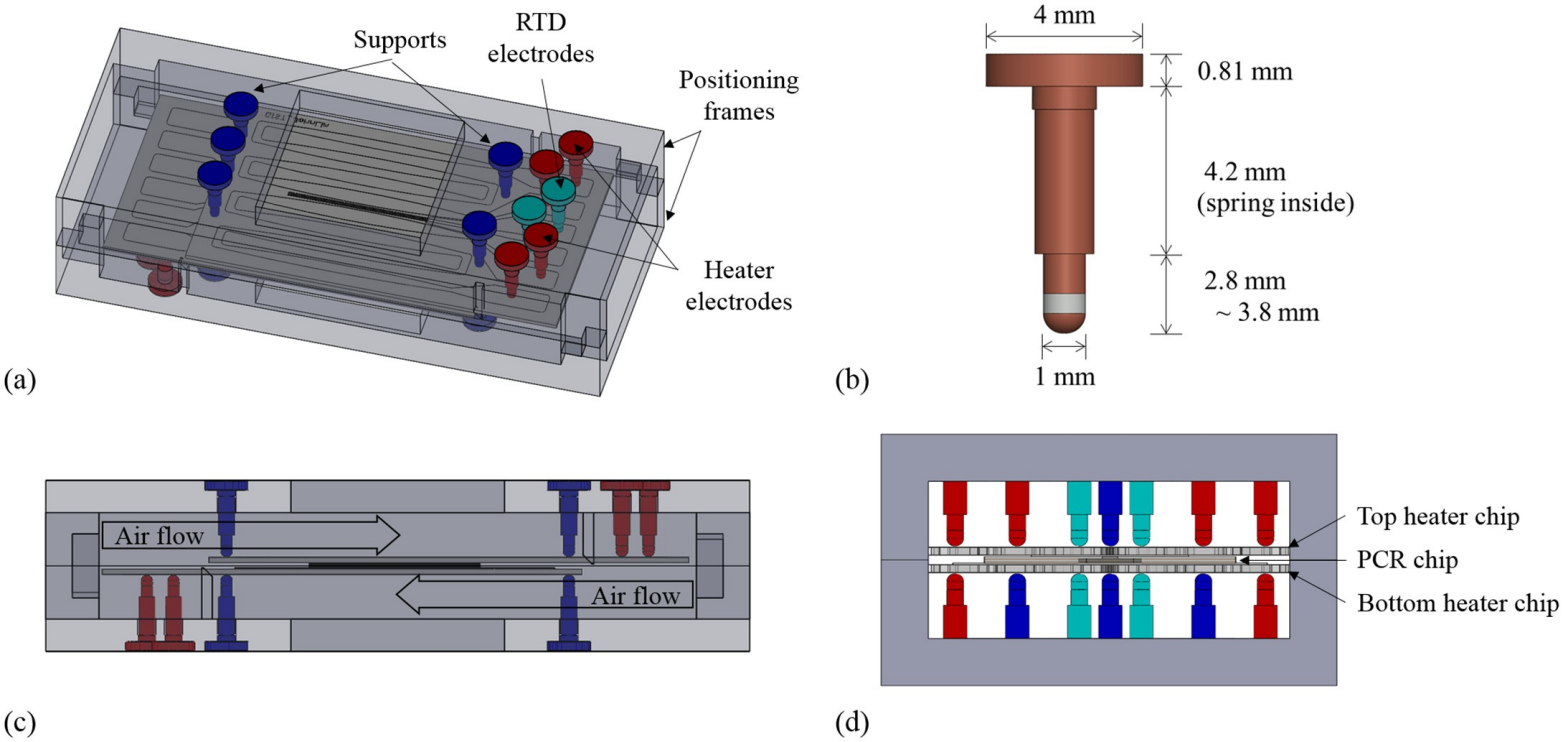
Three-dimensional schematic of the conduction module including dual heater chips, a PCR chip, pin-type electrodes, and positioning frames (a), pin-type electrode with a spring (b), front-view image describing the direction of air flow from the dual fans (c), and side-view image (d).

Fig 4 describes a three-dimensional schematic of the conduction module. As shown in Fig 4(a) and 4(b), pin-type electrodes (0997-5-50-20-75-14-11-0 spring-loaded connector, Mill-Max Manufacturing Corp.) fixed in the positioning frame were used for electrical connection to minimize conduction-induced heat loss. The pin-type electrode with a Cu-coated tip has a spring inside; thus, a stable contact is possible by applying constant pressure to the heater chip. As shown in Fig 4(c) and 4(d), the electrode supports the heater chip both from the top and bottom, allowing the heater chip to float in the air with a minimized physical contact.

The proposed method using pin-type electrodes is advantageous in that the heat loss can be reduced because of a point contact, as compared to when the heater chip makes a surface contact with the positioning frame. Additionally, the pressure applied by the spring induces a tight contact between the heater chip and the PCR chip, increasing the heat transfer efficiency. This mechanism using spring pressure helps the PCR chip to be easily attached and detached to/from the heater chip, as compared to other researches where the heater chip and the PCR chip are bonded with epoxy [12].

The Peltier chip has the disadvantage of high power consumption though the cooling speed is large via conduction [24]. Unlikely, the dual cooling fan (50 mm × 50 mm × 15 mm, BF-0.06A 5015S Blower 12V-2) cools the heater chip through convection, which is slightly slower, but consumes less power (0.63 W). However, if a single fan cools the one side of the PCR chip, a temperature gradient occurs and leads to poor temperature uniformity of the PCR chip. In this study, as shown in Fig 4(c) and 4(d), the temperature gradient is minimized and the uniformity is improved by symmetrical cooling both sides of the heater chip with dual fans. Each fan blows air to the heater in opposite directions; one fan cools the top heater and another cools the bottom heater. The design above enables effective thermal cycling with low power.

## Results and discussion

### Temperature distribution of the PCR chip

**Fig 5:**
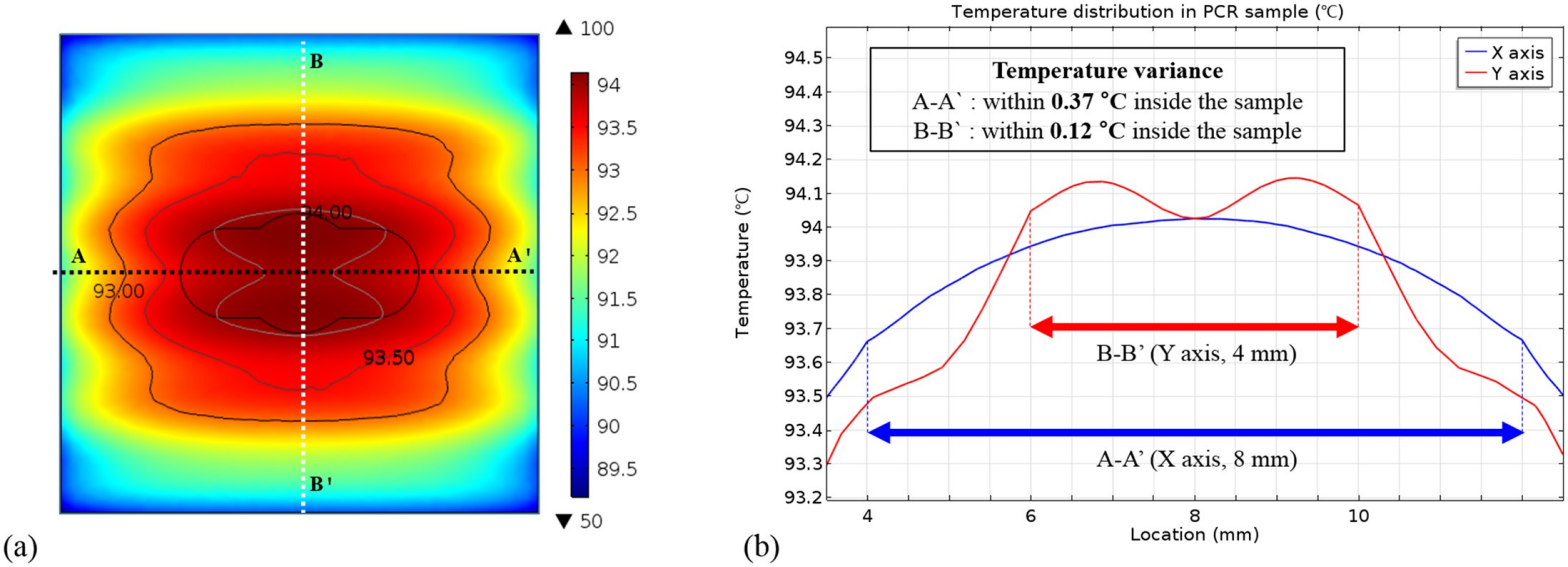
Simulated temperature distribution of the PCR chip by COMSOL Multiphysics.

The temperature distribution in the PCR chamber was calculated using commercial finite element method software (COMSOL Multiphysics, COMSOL Inc., Los Angeles, CA, USA). The computational domain of this simulation includes the Pt heater chip, PCR chip, and PCR sample, whose thermal properties are listed in Table 2. As a result of the calculation shown in Fig 5, the variance of the temperature in the X and Y-axis directions was as small as 0.37 °C and 0.12 °C, respectively, when the target temperature was set to the denaturing temperature (94 °C).

**Table 2:** Thermal properties of the materials used in the simulation of the temperature distribution.

	Pt	Quartz	PI	PET	PCR sample*
Thickness [mm]	0.0002	0.5	0.1	0.1	0.3
Heat capacity [J/Kg K)]	133	730	1100	1000	4180
Thermal conductivity [W/(m K)]	71.6	1.4	0.15	0.15	0.597
Resistivity [Ωm]	4.2 x 10^−7^	−	−	−	−
Ambient temperature [°C]	20				
Air convection coefficient [W/m^2^K]	5				

*In the simulation, the thermal properties of water were be used as for the PCR samples.

### Fabrication

**Fig 6:**
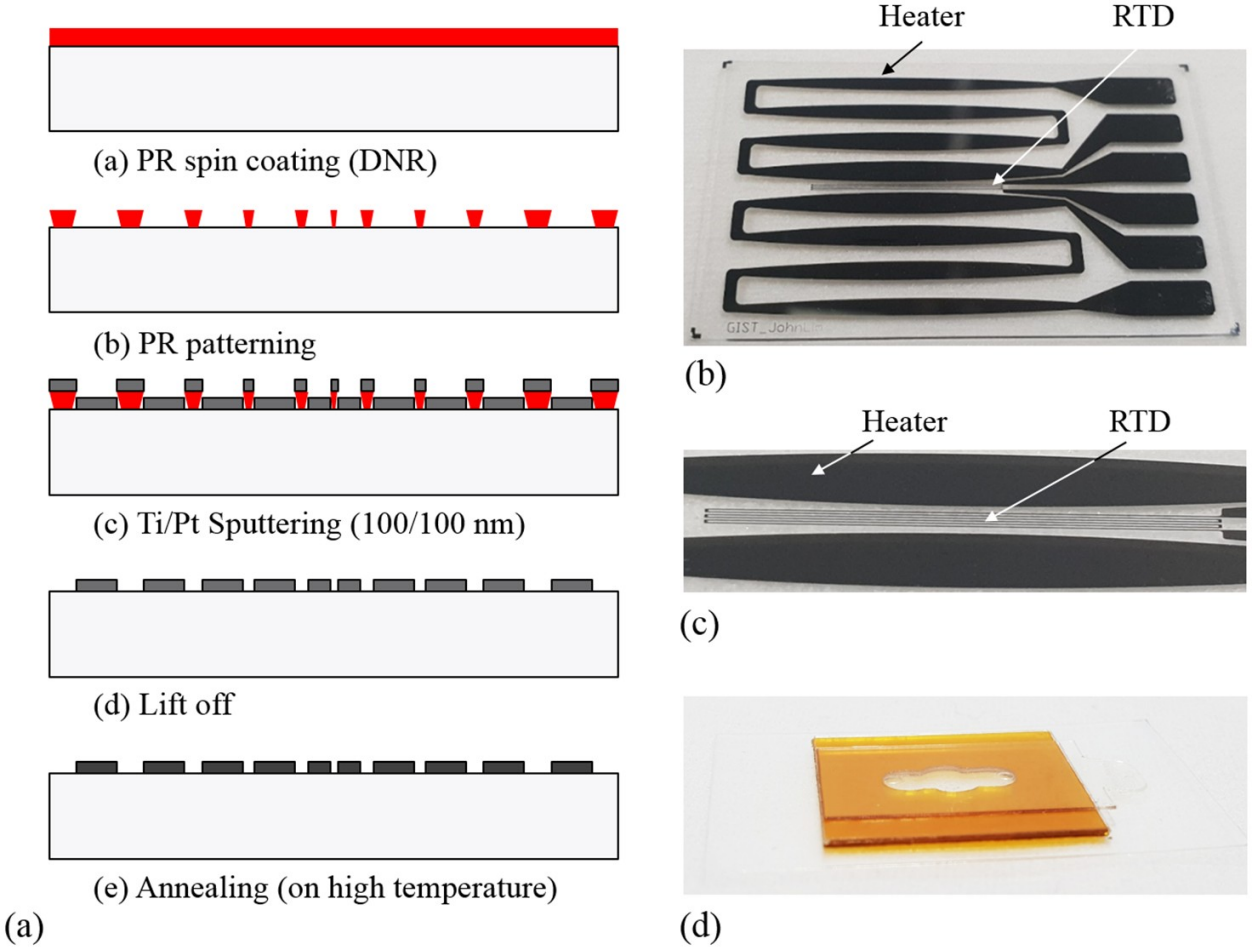
Fabrication sequence of the heater chip with an RTD (a), fabricated heater chip (b), enlarged view of the Pt RTD (c), and fabricated PCR chip (d).

A heater chip can be fabricated, as shown in Fig 6(a), and its sequence is as follows.

1. The heater pattern is intagliated by photoresist (PR) spin coating and lithography on a quartz substrate (26 mm × 45 mm).
2. Ti (100 nm) is deposited as an adhesion layer by RF sputtering, and Pt (100 nm) is deposited by DC sputtering.
3. The Pt heater chip and the RTD are fabricated simultaneously in the lift-off process.
4. The annealing process is performed in N_2_ environment by rapid temperature annealing (RTA) to improve the resistance stability of the RTD.

**Fig 7:**
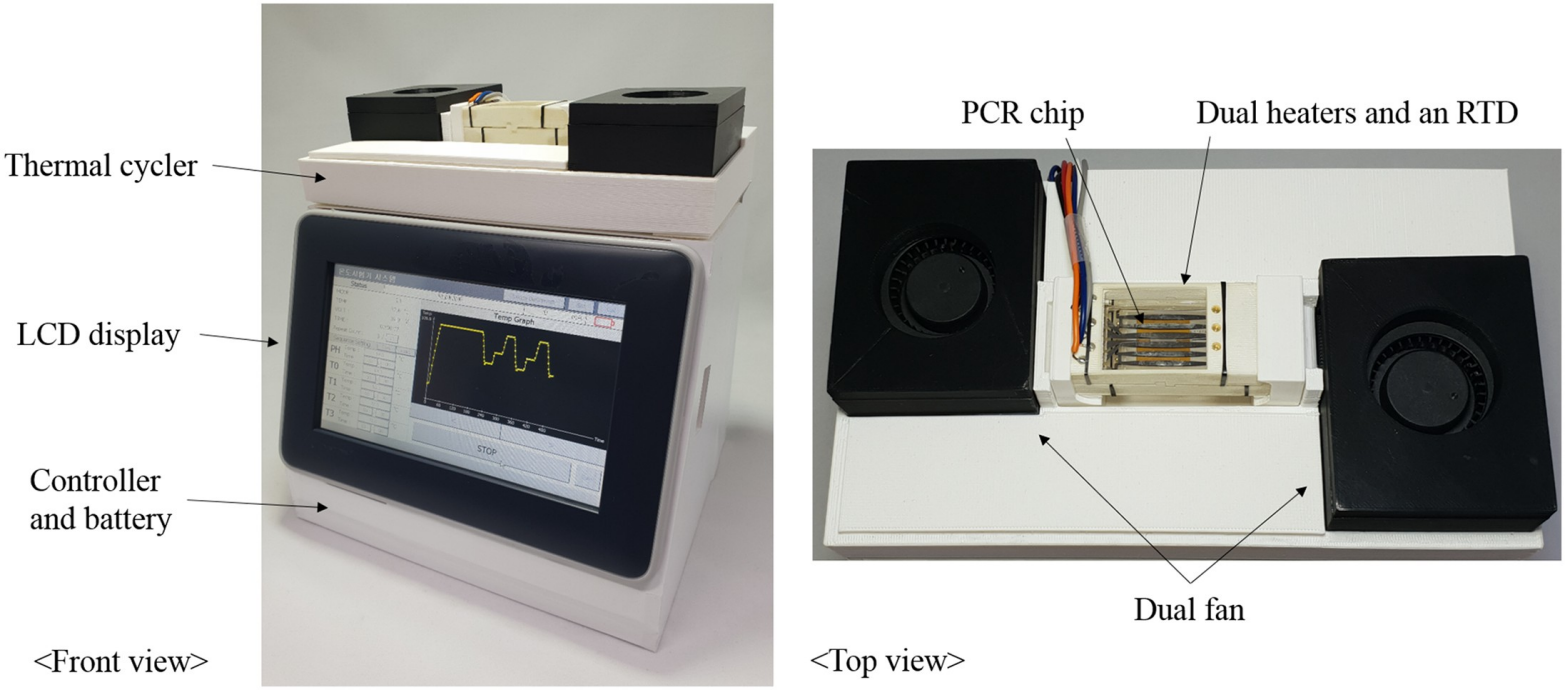
Entire PCR system with dual Pt heaters, dual fans, a stabilized Pt RTD, a controller, and a battery (total size: 210 mm × 170 mm × 230 mm).

The fabricated heater and the RTD are shown in Fig 6(b) and 6(c), and the appearance of the PCR chip made by a cutting plotter is shown in Fig 6(d). As shown in Fig 7, the portable PCR system consists of dual Pt heaters, dual fans, a stabilized Pt RTD, a controller, and a battery. The total system size is 210 mm × 170 mm × 230 mm, and the weighs are about 1.7 kg. The size of the entire system was designed taking into account of a real-time detection module under development at this moment.

### Limitation of the non-annealed Pt RTD

**Fig 8:**
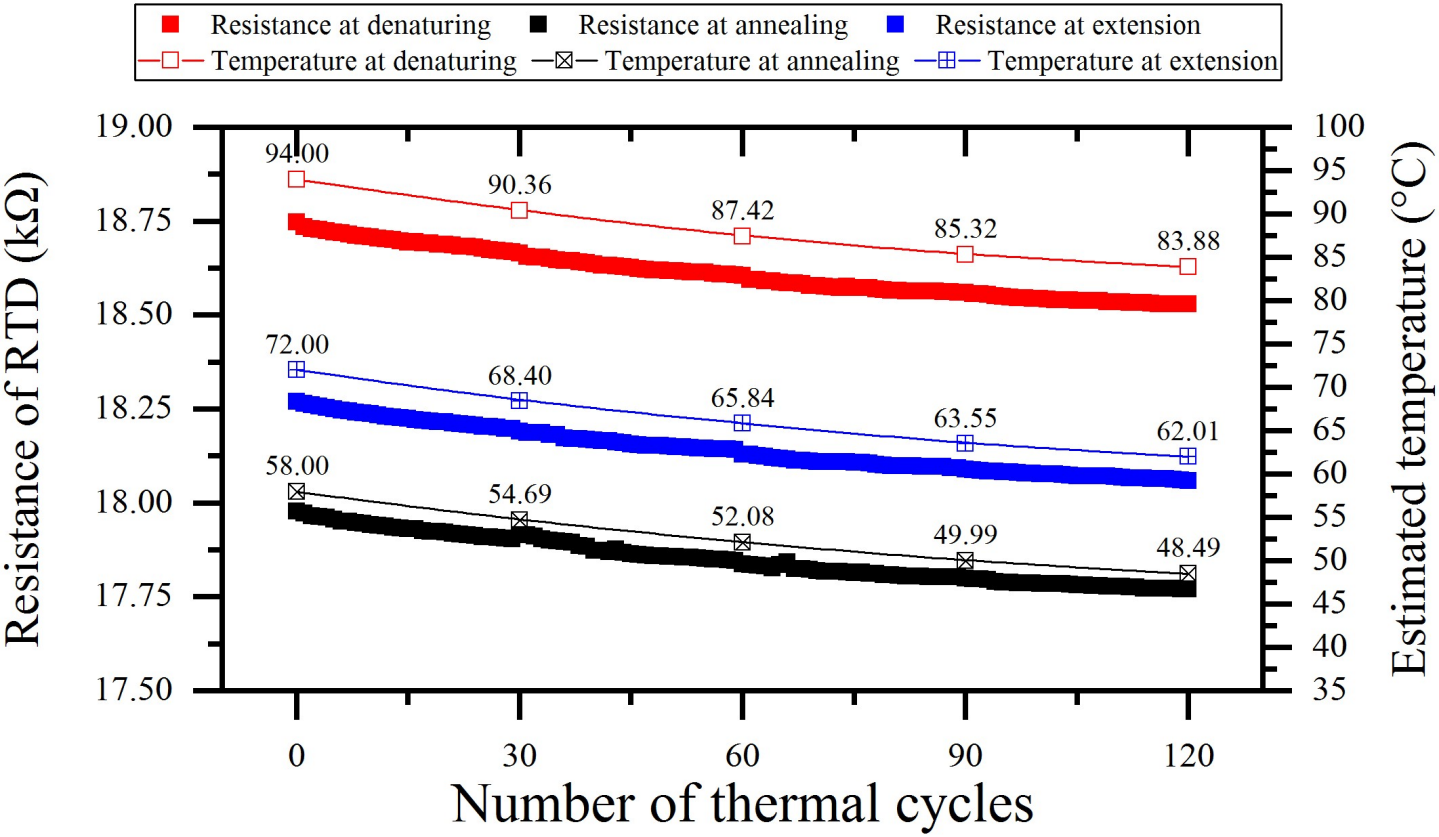
Resistance degradation of the non-annealed Pt RTD during four times of the PCR operations.

In order to investigate the stability degradation in the temperature measurement, thermal cycling was performed with the non-annealed Pt RTD. Fig 8 shows the resistance drop of the RTD, which was measured at every PCR step (denaturing, annealing, and extension) during four times of the PCR operations. As the PCR steps were repeated 30 times in one PCR operation, the resistance was measured 120 times for consecutive monitoring.

Table 3 shows the reduced resistance of the RTD and the equivalent temperature measurement errors (ΔT) between the measured temperature (using a thermocouples) and estimated temperatures (using Pt RTD). The first column of Table 3 shows the temperature-resistance relationship calibrated using thermocouples in the heating equipment. The relationship between temperature and resistance was used to estimate the temperature from the measured resistance of non-annealed Pt RTD. The temperature difference (ΔT) should be 0.5 °C or smaller during one PCR operation to amplify the target DNA successfully. The experimental results show that ΔT tends to be larger at high temperature in denaturing than that at low temperature in annealing. As the number of the PCR operations increases, ΔT decreases gradually, but remains at 1.5 °C even after four times of the PCR operations. Thus, in order to perform the PCR using the non-annealed Pt RTD, stabilization of the resistance should be carried out over a long period of time (more than 10 times of the PCR operations). When the PCR is operated 10 times or more, the ΔT value becomes 0.5 °C or smaller, which allows the thermal cycler to perform the PCR properly. However, as the resistance decrease of the Pt RTD occurs continuously leading to accumulation of the measurement error, there is still the inconvenience that the calibration is required prior to each PCR operation. In this study, an annealing method was proposed to stabilize the resistance of the Pt RTD for the PCR system using RTA [21, 31].

**Table 3:** Temperature measurement error caused by resistance degradation of the non-annealed Pt RTD during four times of the PCR operations.

Number of PCR operations	Temperature-resistance relationship			1			2			3			4		
PCR steps	[°C]	Resistance [kΩ]		Resistance (kΩ)	°C		Resistance (kΩ)	°C		Resistance (kΩ)	°C		Resistance (kΩ)	°C	
	T_TC_ (= T_e(0)_)				T_e(1)_	ΔT_(1)_		T_e(2)_	ΔT_(2)_		T_e(3)_	ΔT_(3)_		T_e(4)_	ΔT_(4)_
Denaturing	18.75	94	−	18.67	90.36	-3.64	18.61	87.42	-2.94	18.56	85.32	-2.10	18.53	83.88	-1.45
Extension	18.27	72	−	18.20	68.40	-3.60	18.14	65.84	-2.56	18.09	63.55	-2.28	18.06	62.01	-1.54
Annealing	17.98	58	−	17.90	54.69	-3.31	17.85	52.08	-2.61	17.80	49.99	-2.10	17.77	48.49	-1.49

T_TC_: temperature measured by thermocouples, T_e_: estimated temperature, ΔT: temperature measurement errors (ΔT_(i)_ = T_e(i)_ − T_e (i-1)_)

### Enhanced stability of the Pt RTD after the annealing process

Annealing at a temperature higher than the denaturing temperature greatly reduces the resistance of the RTD via recrystallizing the Pt thin film, thereby preventing additional resistance reduction during the PCR operations. Another major condition in annealing would be its duration. Table 4 shows the annealing conditions and the average values of the resistance drop rate of each Pt element after the annealing. The annealing process at high temperature and/or long time tends to lead to a high drop in the resistance (*ΔR*).

**Table 4:** Various conditions of annealing.

	Annealing condition		Resistance drop rate [%] (= *Δ* R / initial R)			
Condition No.	Temperature (°C)	Time (min)	Pt heater 1	Pt heater 2	Pt RTD	Average
1	450	2	23.3	24.0	24.7	24.0
2	450	5	26.0	26.2	28.0	26.7
3	600	2	30.7	30.4	32.2	31.1
4	600	5	35.8	38.0	39.1	37.6
5	750	2	34.8	35.3	35.5	35.2
6	750	5	35.1	34.9	37.7	35.9

As shown in Table 4, under the condition of 2 min annealing, the resistance drop rate of the Pt elements was found to increase with the annealing temperature. However, under the condition of 5 min annealing, the resistivity drop rate was the highest at 600 °C and slightly lower at 750 °C. The reduced drop rate at 750 °C can be explained by the so-called island formation where pores are formed on the surface of the thin film while Ti and Pt particles form clusters in the annealing process [31].

**Fig 9:**
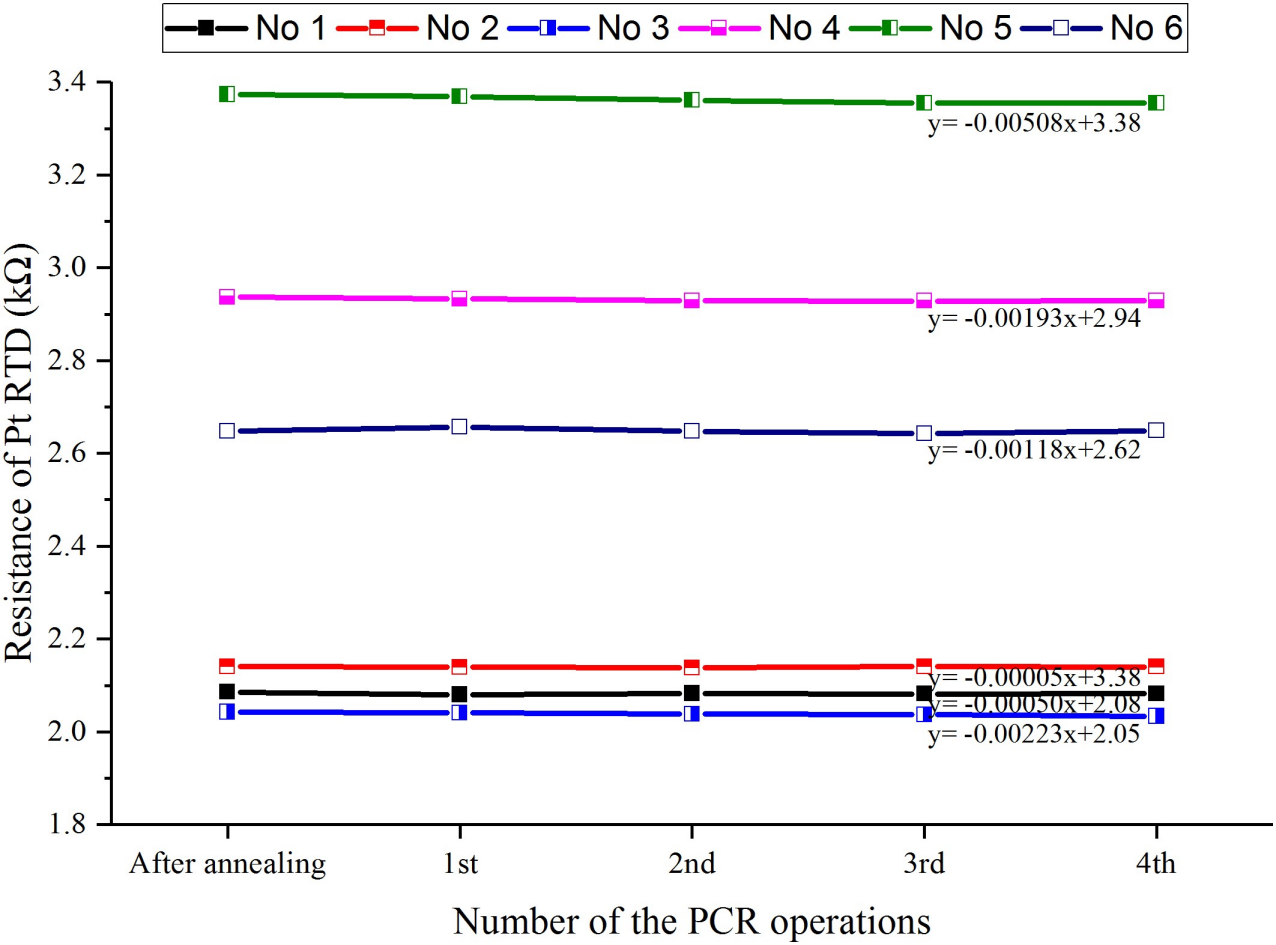
Resistance change versus number of the PCR operations.

After annealing the Pt RTD, another resistance-temperature relationship was acquired by the same calibration procedure as used for the non-annealed Pt RTD. Then the resistance stability was confirmed by measuring the resistance in the PCR operations repeated four times (Fig 9). The analysis of the measured resistance indicates that the stability of the RTD is higher when the resistance drop is smaller after several PCR operations. The stability of the RTD was greatly enhanced with condition 2 (450 °C, 5 min), whose resistance drop was measured to be as small as 0.005% (equivalently to 0.044 °C in the temperature error). With annealing condition 5, in which the improvement in the stability of the RTD was the worst, the resistance drop was 0.5% (equivalently to 4.4 °C in the temperature error). It can be seen that the stability of the RTD is drastically improved under all annealing conditions considering that the resistance drop of the non-annealed RTD is at least 1.14% (equivalently to 10.02 °C in the temperature error).

### PCR performance

The PCR was performed in a laboratory circumstance with the following conditions, as shown in Table 5. The temperature, time, and number of cycles of the PCR operation can be adjusted via the LCD touch screen. The temperature of the PCR chip is controlled within ± 0.5 °C by the control board, whose progress can be monitored through the LCD touch screen. The PCR operation is completed within 70 min when all 30 cycles are carried out. The reduced thermal mass of the heater chip enhanced the heating and cooling rates by 1.77 and 1.43 times, respectively, as compared to that of the previous work [10]. The enhanced thermal cycling speed results in shortened total PCR time by 12 min.

**Table 5:** PCR conditions and thermal cycling speed.

PCR condition			Thermal cycling speed		
–	Pre-denaturing step	94 °C / 180 s	2.88 °C/s	25 °C—94 °C	(24 s)
30 Cycles	Denaturing step	94 °C / 30 s	1.29 °C/s	72 °C—94 °C	(28 s)
	Annealing step	58 °C / 30 s	1.75 °C/s	94 °C—58 °C	(8 s)
	Extension step	72 °C / 30 s	2.00 °C/s	58 °C—72 °C	(11 s)
–	Post-extension step	72 °C / 180 s	–		

The heater chip with a reduced area decreases not only the duration time of the thermal cycling, but also the power consumption. The PCR system using a Pt thin-film heater and dual fans consumes only 4.11 W and 0.63 W for heating and cooling, respectively, whereas a commercial PCR equipment using a Peltier chip consumes more than 500 W [1]. The total power consumption for one PCR operation was measured to be as small as 3.2 Wh, which is equivalent to 21% of that in the previous work (15 Wh) [10]. More specifically, a power meter (Electrical Parameter Tester, 33V3A) analyzed that the thermal cycler (heater and cooler) consumes 1.53 Wh, whereas the LCD touch screen consumes 1.57 Wh. The portability of the PCR system can be further enhanced with a real-time detection module. Even if the detection module is embedded into the PCR system in near future, it is still possible to operate the system with the battery. Portable PCR operation can be performed more than four times since the expected power consumption of the detection module is less than 2.44 Wh [11]. The total power consumption can be reduced further by replacing the LCD touch screen with a low-power display. However, the power consumption of the thermal cycler is still slightly higher than that of other low-power PCR systems [12]. One of the effective approaches to reduce the power consumption is the reduction of the area of the heater chip. However, this tends to degrade temperature uniformity over a certain area, which will not allow the PCR chip to be used for multiwell PCR. Therefore, it is important to maintain temperature uniformity in a certain area even when using multiwell PCR chips later, rather than reducing the thermal mass of the heater chip. The optimal electrode shape in the heater chip resulted in a slight improvement in the temperature uniformity, as compared to the previous work [10], despite the reduced size of the heater chip.

*Escherichia coli* (*E*. *Coli*) bacteria, which are commonly used to verify the PCR performance, were used in this study. A total of 80 μl of the PCR sample was prepared by mixing *E*. *Coli* genomic DNA (4 μl, 100 ng/μl), forward primer (4 μl, 10 pmol/μl), reverse primer (4 μl, 10 pmol/μl), double-distilled water (28 μl), and PCR mix (40 μl, Labchip 2X premix). The forward primer (5’-ATAAATCGCCATTCGTTGACTAC-3’) and reverse primer (5’-AGAACGCCCACTGAGATCATC-3’) were used to amplify 180 base pair of product. It is recommended that the PCR chamber (10 μl) is not completely filled with PCR sample during an injection process. Otherwise, the PCR sample can overflow through inlet/outlet holes when the cover layer is attached to seal the PCR chamber. Overflow of the sample can cause poor adhesion between the cover layer and the chamber layer, which leads to severe evaporation of the sample during PCR operations. The injected sample volume into the film-type PCR chip was 8.5 μl (8.5 μl, PLOS one 8 μl), whereas the conventional PCR system (A200, LongGene Scientific Instruments Co., Ltd., China) required to fill the vial tube with 20 μl of the sample at least.

**Fig 10:**
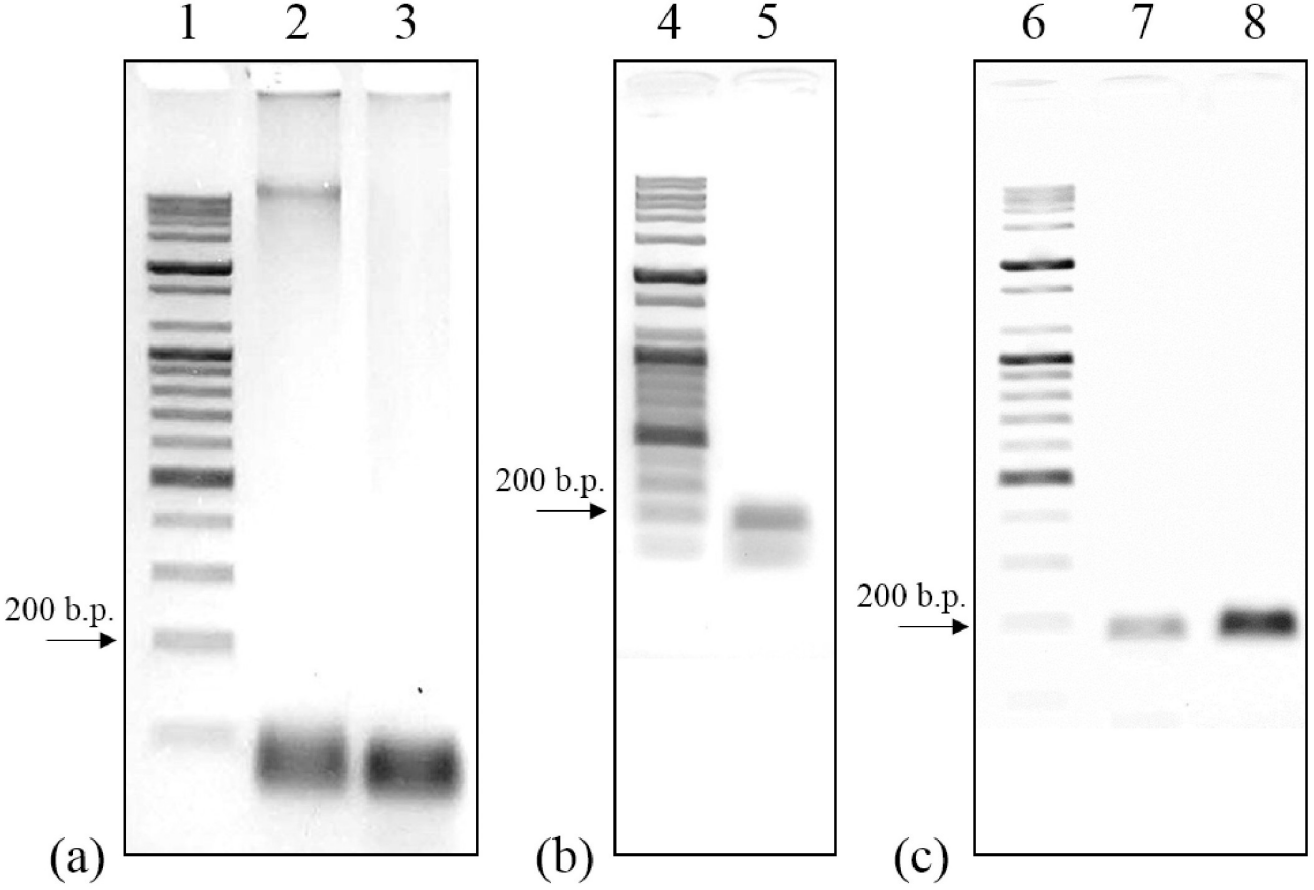
Gel-electrophoresis result of the amplified genomic DNA band of E. Coli bacteria. The electrophoresis result of the PCR using the non-annealed RTD (a), annealed RTD (b), and comparison with the conventional PCR system (c); Lanes 1, 4, and 6: ladder marker, Lanes 2 and 3: thin-film heater with the non-annealed RTD, Lane 5: thin-film heater with the annealed RTD with high annealing temperature (60 °C), Lane 7: Pt thin-film heater and RTD PCR system, and Lane 8: conventional PCR system.

Fig 10 shows the gel-electrophoresis results of the amplicon after the PCR operations. The detailed PCR conditions are listed in Table 6. Lanes 1, 4, and 6 are the DNA marker ladder (100 bp Plus DNA ladder, Bioneer, South Korea) for indicating the length (base pair) of the amplicon. As shown in lane 2 of Fig 10(a), the PCR operation using the non-annealed RTD shows unsuccessful amplification result, in which a smearing band and a nonspecific band appeared. The smearing band in lane 2 was generated by the low denaturing temperature (90 °C), which lead to incomplete denaturation of genomic DNA [32]. In the consecutive experiment (lane 3), the smearing band disappeared. This is because the denaturing temperature was increased as the resistance of the non-annealed Pt RTD decreased during the PCR operation. A nonspecific band shorter than the target base pair (180 bp) was generated because of the high annealing temperature. The gel-electrophoresis results in lane 5 of Fig 10(b) confirm this phenomenon experimentally. The nonspecific band (lane 5) appeared when the annealing temperature was set to be higher (60 °C) than the appropriate annealing temperature (58 °C).

**Table 6:** PCR conditions of the individual lanes in gel-electrophoresis.

Lane number	1	2	3	4	5	6	7	8
PCR system	Marker ladder	Portable PCR	Portable PCR	Marker ladder	Portable PCR	Marker ladder	Portable PCR	Conventional PCR
PCR chamber	−	Thin film PCR chip	Thin film PCR chip	−	Thin film PCR chip	−	Thin film PCR chip	Vial tube
Temperature sensor	−	Non-annealed RTD	Non-annealed RTD	−	Annealed RTD	−	Annealed RTD	Thermo-couple
Temperature setting	−	94 °C (30s) 58 °C (30s) 72 °C (30s)	94 °C (30s) 58 °C (30s) 72 °C (30s)	−	94 °C (30s) 60 °C (30s) 72 °C (30s)	−	94 °C (30s) 58 °C (30s) 72 °C (30s)	94 °C (30s) 58 °C (30s) 72 °C (30s)
Remarks	−	Failure in denaturing / nonspecific band	Nonspecific band	−	Nonspecific band	−	Weak intensity	−

The amplification result (lane 7) of the portable PCR system using the annealed Pt RTD was compared with that of the conventional PCR system (lane 8) in Fig 10(c). The target base pair of *E*. *coli* genomic DNA was successfully amplified with a distinct band. The intensity of the amplified genomic DNA (lane 7) is slightly lower compared to that of the conventional PCR system (lane 8). This can be explained by the fact that the amplification efficiency of PCR tends to increase as the surface to volume ratio (SVR) decreases; the SVR of the film-type PCR chip is higher than that of vial tube. Thus, using a film-type PCR chip may result in slightly low amplification efficiency. In conclusion, it can be seen that the PCR performance is reasonable considering the characteristics of portable PCR systems, which are operable with low power using film-type PCR chips. In addition, the PCR system can be used in outdoor environments even when the temperature varies out of the operable temperature range of thermocouples (15–35 °C).

## Conclusion

In this study, a Pt heater chip with a Pt RTD and dual fans were used for a portable PCR system. As a Pt RTD can measure the absolute temperature and is less affected by the outdoor temperature, it is suitable for outdoor PCR systems, and its stability as a temperature sensor was greatly improved through the annealing process. The Pt heater chip was fabricated on a quartz plate that can withstand the annealing temperature. The reduced thermal mass of the heater chip and PCR chip allows performing the PCR with low power consumption. The target DNA was stably amplified using the fabricated PCR chip, heater chip, and thermal cycler. The amplification result of the PCR system was compared with that of conventional PCR using a vial tube through gel-electrophoresis. As a result, although the PCR was performed with low power consumption, the band of the amplicon appeared clearly, but with slightly lower intensity, as compared to that using the commercial device. Therefore, the PCR system with the Pt RTD is suitable for outdoor use or small unmanned vehicles, which is inferred from the experimental results. For the improvement of the PCR system, it is necessary to reduce the actual power consumption of the PCR system by replacing the present touch screen with a low-power display, and to simultaneously detect multiple samples using a multiwell PCR chip.

## Supporting information

S1 Table Resistance drop rate of Pt RTD after the annealing process. (PDF)

S2 Table Resistance change of thin film Pt heater chip with a RTD through PCR operations. (PDF)

S1 Figure Measured temperature during PCR operation with non-annealed Pt RTD. (PDF)

S2 Figure Description for a phenomenon that the temperature of non-annealed Pt RTD increases as the resistance of RTD decreases. (PDF)

S3 Figure Averaged resistance drop rate of Pt RTD after the annealing process. (PDF)
