# Supplementary material for: Battery-operated portable PCR system with enhanced stability of Pt RTD
Source: PLoS One. 2019 Jun 27;14(6):e0218571. doi: 10.1371/journal.pone.0218571 (PMC6597155; doi:10.1371/journal.pone.0218571)
Supplement: S1 Table — (PDF) [file pone.0218571.s001.pdf]

S1 Table. Resistance drop rate of Pt RTD after the annealing process.

Annealing condition and resistance drop rate (%)

2 min	heater 1	heater 2	Sensor	averaged rate
450℃	22.78	23.80	24.36	23.64
600℃	31.03	30.20	32.15	31.13
750℃	34.92	35.28	35.34	35.18
5 min	heater 1	heater 2	Sensor	averaged rate
450℃	26.62	26.56	27.88	27.02
600℃	36.09	38.16	39.02	37.76
750℃	35.06	34.94	37.74	35.91
