# Supplementary material for: Battery-operated portable PCR system with enhanced stability of Pt RTD
Source: PLoS One. 2019 Jun 27;14(6):e0218571. doi: 10.1371/journal.pone.0218571 (PMC6597155; doi:10.1371/journal.pone.0218571)
Supplement: S2 Table — (PDF) [file pone.0218571.s002.pdf]

S2 Table. Resistance change of thin film Pt heater chip with a RTD through PCR operations.

Heater Chip		Annealing condition		Resistance		Drop rate (%)	Number of PCR operations							
		Temp. (℃)	Time (min)	Before annealing	After annealing		1st	(%)	2nd	(%)	3rd	(%)	4th	(%)
No 1	Heater 1 (Ω)	450	2	159	123	22.7799	122.60	-0.15	122.83	0.19	122.30	-0.43	122.69	0.32
	Heater 2 (Ω)			154	117	23.7987	117.21	-0.12	117.42	0.18	116.86	-0.48	117.43	0.49
	Pt Sensor (kΩ)			2.7570	2.0855	24.3562	2.0799	-0.27	2.0825	0.13	2.0818	-0.03	2.0820	0.01
No 2	Heater 1 (Ω)	450	5	169	124	26.6154	123.96	-0.05	123.85	-0.09	124.79	0.76	124.28	-0.41
	Heater 2 (Ω)			168	123	26.5595	123.05	-0.27	123.03	-0.02	123.98	0.77	124.07	0.07
	Pt Sensor (kΩ)			2.9680	2.1404	27.8841	2.1394	-0.05	2.1378	-0.07	2.1407	0.14	2.1395	-0.06
No 3	Heater 1 (Ω)	600	2	166	114	31.0301	113.99	-0.44	112.99	-0.88	113.12	0.12	112.22	-0.80
	Heater 2 (Ω)			168	117	30.2024	116.29	-0.83	115.20	-0.94	116.64	1.25	116.20	-0.38
	Pt Sensor (kΩ)			3.0100	2.0424	32.1462	2.0409	-0.07	2.0382	-0.13	2.0370	-0.06	2.0332	-0.19
No 4	Heater 1 (Ω)	600	5	268	171	36.0858	170.91	-0.22	171.20	0.17	171.48	0.16	171.41	-0.04
	Heater 2 (Ω)			297	184	38.1650	183.42	-0.13	183.55	0.07	183.69	0.08	183.88	0.11
	Pt Sensor (kΩ)			4.8160	2.9366	39.0241	2.9325	-0.14	2.9290	-0.12	2.9287	-0.01	2.9288	0.01
No 5	Heater 1 (Ω)	750	2	302	197	34.9238	195.80	-0.37	196.89	0.56	195.17	-0.87	195.68	0.26
	Heater 2 (Ω)			266	172	35.2782	171.91	-0.15	171.62	-0.17	170.61	-0.59	171.59	0.58
	Pt Sensor (kΩ)			5.2170	3.3732	35.3422	3.3691	-0.12	3.3612	-0.23	3.3549	-0.19	3.3549	0.00
No 6	Heater 1 (Ω)	750	5	251	163	35.0598	163.00	0.00	163.00	0.00	162.70	-0.18	163.16	0.28
	Heater 2 (Ω)			249	162	34.9398	162.00	0.00	162.00	0.00	161.30	-0.43	161.76	0.29
	Pt Sensor (kΩ)			4.2530	2.648	37.7381	2.6568	0.33	2.6480	-0.33	2.6430	-0.19	2.6490	0.23
