# Supplementary figures and images for: Battery-operated portable PCR system with enhanced stability of Pt RTD
Source: PLoS One. 2019 Jun 27;14(6):e0218571. doi: 10.1371/journal.pone.0218571 (PMC6597155; doi:10.1371/journal.pone.0218571)

S1 Figure. Measured temperature during PCR operation with non-annealed Pt RTD.

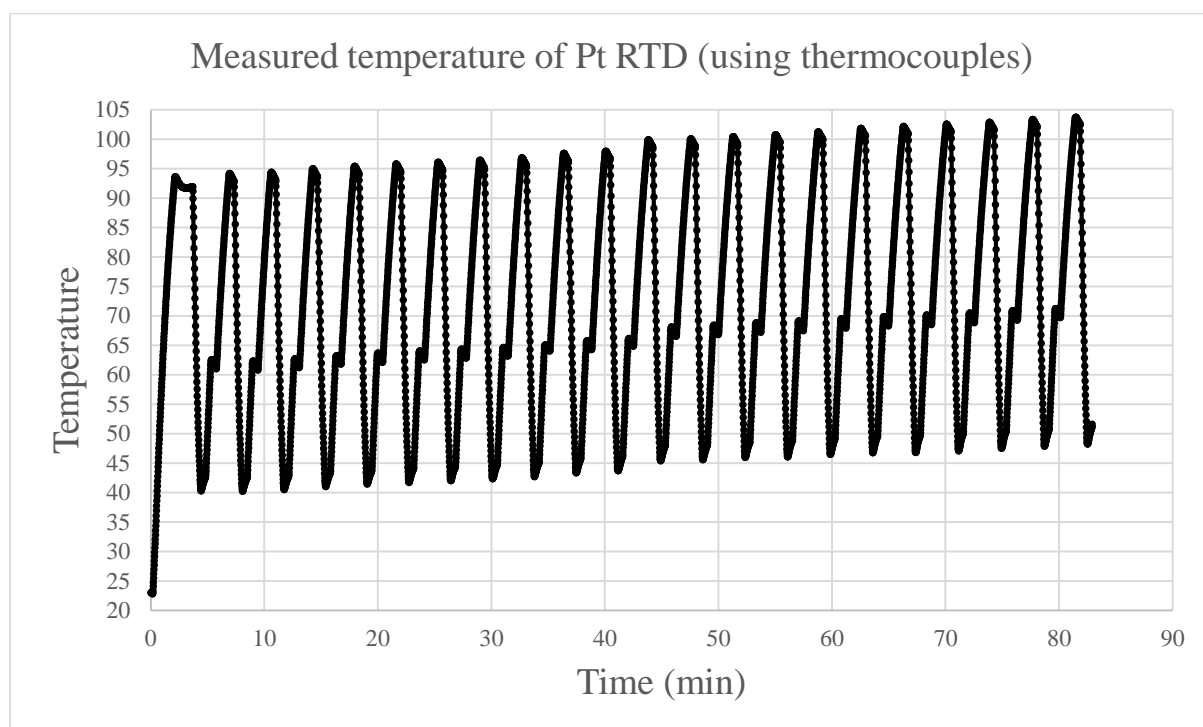

Supplement: S1 Figure — (PDF) [file pone.0218571.s003.pdf]

S3 Figure. Averaged resistance drop rate of Pt RTD after the annealing process.

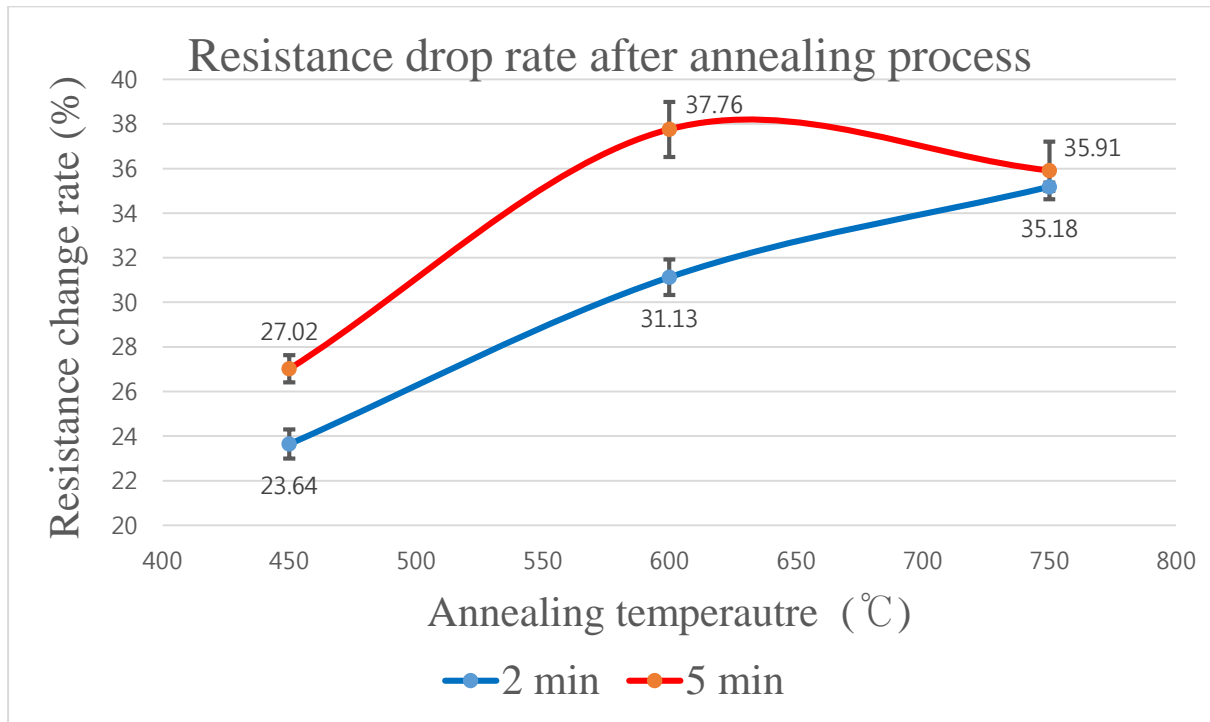

Supplement: S3 Figure — (PDF) [file pone.0218571.s005.pdf]
