# Supplementary material for: Battery-operated portable PCR system with enhanced stability of Pt RTD
Source: PLoS One. 2019 Jun 27;14(6):e0218571. doi: 10.1371/journal.pone.0218571 (PMC6597155; doi:10.1371/journal.pone.0218571)
Supplement: S2 Figure — (PDF) [file pone.0218571.s004.pdf]

S2 Figure. Description for a phenomenon that the temperature of non-annealed Pt RTD increases as the resistance of RTD decreases.

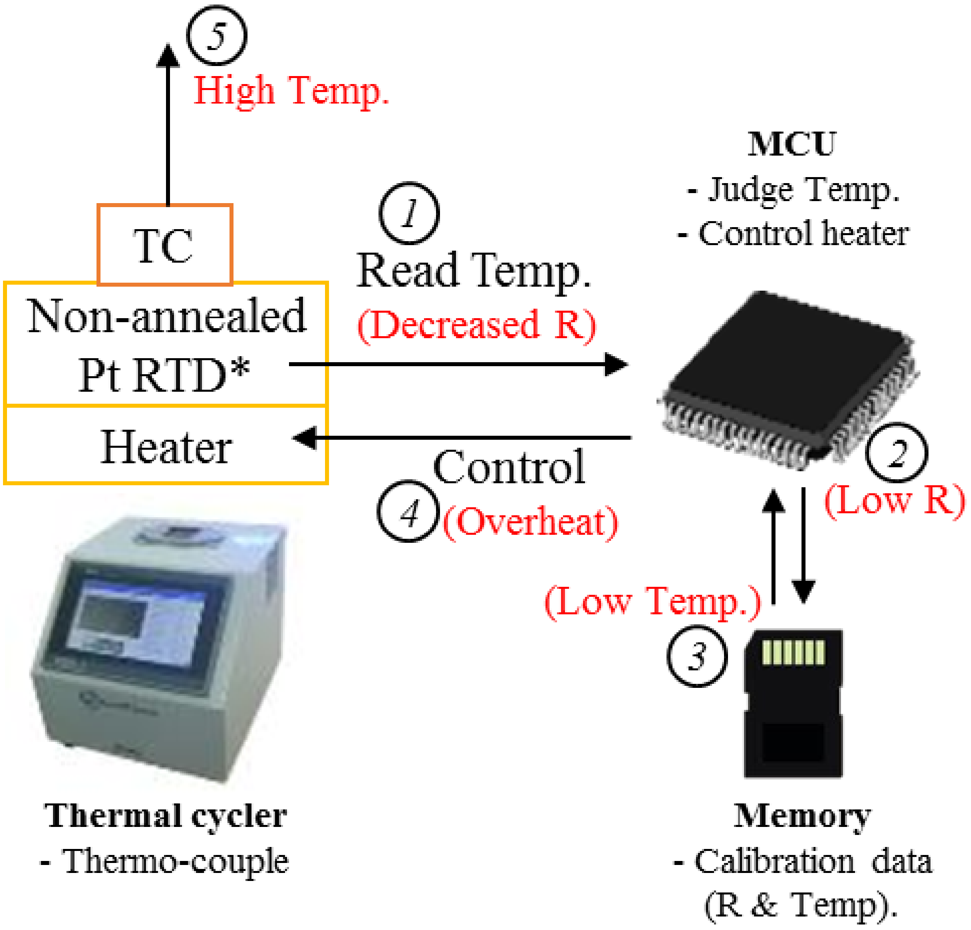

*Resistance of Pt RTD tends to decrease during thermal cycle, especially in case of thin film.
